# Ethical considerations of gene editing and genetic selection

**DOI:** 10.1002/jgf2.321

**Published:** 2020-05-29

**Authors:** Jodie Rothschild

**Affiliations:** ^1^ Rothschild Biomedical Communications Seattle WA USA

**Keywords:** ethical, ethics, gene editing, genetic selection

## Abstract

For thousands of years, humans have felt the need to understand the world around them—and ultimately manipulate it to best serve their needs. There are always ethical questions to address, especially when the manipulation involves the human genome. There is currently an urgent need to actively pursue those conversations as commercial gene sequencing and editing technologies have become more accessible and affordable. This paper explores the ethical considerations of gene editing (specifically germline) and genetic selection—including the hurdles researchers will face in trying to develop new technologies into viable therapeutic options.

## 1 BACKGROUND

### 1.1 Gene editing

Artificial manipulation of genes is a relatively new science, and a number of watershed moments have provided the foundation for the current state of genetic engineering. Researchers first discovered that nonspecific alterations to *Drosophila* DNA could be introduced using radiation^1^ and chemicals^2^ in 1927 and 1947, respectively. Greater understanding of the structure of the DNA molecule (such as the work of Watson, Crick, and Franklin, leading to the discovery of DNA’s double‐helix structure^3^) and the cellular processes that govern its transcription, translation, replication, and repair (such as the function of ligases^4^ and restriction enzymes^5^) led to the first splicing experiments^6^ and, ultimately, the first recombinant DNA^7^ in the early 1970s. DNA recombination techniques were used extensively in the budding yeast *Saccharomyces cerevisiae* ^8^, ^9^ beginning in the early 1980s, allowing researchers to study functional eukaryotic genomics. And in a significant advancement, the development of polymerase chain reaction (PCR) allowed scientists to amplify DNA, producing millions of copies from a single strand.^10^

Around the same time, a number of laboratories created the first transgenic mice,^11^ and about five years later, the first knockout mice were created.^12^ Targeted gene editing was further advanced by the discovery that engineered endonucleases could create site‐specific double‐stranded breaks (DSBs), which in turn induce homologous recombination (HR),^13^, ^15^ the most common type of homology‐directed repair (HDR). When the Human Genome Project was declared complete in 2003,^15^ it became possible to identify (and thus, theoretically, target) any human gene of interest.

The three main techniques for gene editing involve molecules that recognize and bind to specific DNA sequences; researchers can use custom molecules to affect genetic and epigenetic changes on essentially any gene. For example, these molecules can be combined with endonucleases, creating DSBs which can be repaired using either nonhomologous end joining (NHEJ), which often results in small random indel mutations, or HDR, which, when donor DNA with homology to either side of the cleavage site is present, can be used to create new or “repaired” versions of a target gene. The site‐specific DNA recognition molecule can also be combined with an effector molecule to up‐ or downregulate gene expression.

#### 1.1.1 ZFPs/ZFNs

In the late 1970s and early 1980s, there was a large focus on understanding transcription factor IIIA (TFIIIA), the first eukaryotic transcription factor to be described. In 1983, researchers determined that zinc is required for TFIIIA function,^16^ and in 1985 came the discovery that the zinc‐binding portions of the proteins are actually repeating motifs, independently folded to create finger‐like domains that grip the DNA.^17^ This class of proteins is now referred to as zinc finger proteins (ZFPs), and several similar proteins have been discovered in the proteomes of a number of different organisms. Because each zinc finger recognizes three base pairs,^18^, ^19^, ^20^ a peptide can be created to recognize a target gene by joining the appropriate zinc fingers in a linear fashion.

A 1994 paper describes a ZFP that was engineered to recognize and suppress an oncogene, as well as a ZFP that acted (in a different cell system) as a promoter of another gene by recognizing its activation domain.^21^ The same paper suggests that ZFPs can be bound to effector proteins as a means of controlling gene expression.

Building on this idea, researchers fused a ZFP to the nonspecific cleavage domain of the Fok1 restriction enzyme.^22^ The resulting heterodimer, known as a zinc finger nuclease (ZFN), can recognize a specific DNA sequence and produce a targeted DSB. As previously mentioned, these DSB can either be repaired via NHEJ, resulting in small indels, or HDR, which can be harnessed to insert an alternate or repaired gene. Fok1 must dimerize, so ZFNs must be created in pairs (one targeting the 3’ strand and the other targeting the 5’ strand) which improves target specificity—though efficiency remains relatively low (G‐rich sequences are especially difficult to target).

Ex vivo and in vivo delivery of ZFNs is relatively easy given their small size and the small size of the ZFN cassettes (which allows for the use of a variety of vectors). However, while ZFNs were certainly novel at the time they were developed, they are incredibly difficult and expensive to engineer, making them less practical in general than newer technologies.

#### 1.1.2 TALEs/TALENs

In 2009, two different laboratories described a newly identified DNA‐binding motif: the transcription activator‐like effector (TALE), a protein secreted by the plant pathogen *Xanthomonas*.^23^, ^24^ Each TALE includes a DNA‐binding region composed of tandem repeats with repeat‐variable diresidues (RVDs) at positions 12 and 13; each RVD recognizes an individual nucleotide.

Like ZFPs, synthetic TALEs can be designed to affect gene regulation,^25^ combined with effector proteins, or fused to endonucleases^26^, ^27^, ^28^ to create TALE nucleases (TALENs); as with ZFNs, because Fok1 is the endonuclease used, TALENs must be created in pairs.

TALE nucleases are much larger than ZFNs, and so can be more difficult to deliver efficiently (especially in vivo). However, for myriad reasons (including the nature of their relative interactions with the DNA and the fact that each RVD recognizes a single base), TALE‐based chimeras (especially TALENs) can be built with higher specificity and greater targeting capacity than ZFP‐based chimeras. In addition, TALENs can be produced significantly more cheaply, easily, and with greater efficiency than ZFNs.

#### 1.1.3 CRISPR‐Cas

In 1987, a laboratory in Osaka accidentally discovered an unusual palindromic repeat sequence in the *E. coli* genome they were studying, unique in that it was regularly interspaced.^29^ These DNA motifs were further identified in various bacterial genomes by multiple laboratories over the next 20 years; their function, however, was still unknown. By 2005, three groups had independently determined that the spacer sequences were actually derived from phage DNA,^30^, ^31^, ^32^ and the possibility of the genes playing a role in bacterial immunity was first suggested.^30^, ^33^ By this time, the scientific community referred to this unusual array as clustered regularly interspaced short palindromic repeats, or CRISPR. Meanwhile, researchers in the Netherlands had identified several other genes located near the CRISPR locus that appeared to be functionally associated with the CRISPR genes;^34^ these would turn out to be the CRISPR associated proteins (Cas) that make up an integral part of the CRISPR‐Cas system.

In 2007, the CRISPR‐Cas system was identified as being a prokaryotic defense against pathogens.^35^ As a part of a self‐/non–self‐determination mechanism of adaptive immunity, prokaryotes integrate a segment (generally 32‐38 base pairs) of phage DNA into their own genome, creating the spacers in the CRISPR arrays. After the CRISPR genes are transcribed, endoribonucleases cleave the resulting CRISPR RNA (pre‐crRNA), resulting in shorter RNA units composed of a single spacer sequence and the palindromic repeat (crRNA); depending on the organism, a trans‐activating crRNA (tracrRNA) may also be transcribed. The RNA forms a ribonucleoprotein (RNP) complex with the associated Cas proteins; any phage DNA containing the spacer sequence will be identified by the guiding RNA and cleaved by the endonuclease function of the Cas protein(s). The protospacer is the homologous sequence in the invading DNA, and is followed by a short protospacer adjacent motif (PAM); because the PAM is not incorporated in the CRISPR array, the CRISPR‐Cas complex is able to recognize the foreign DNA as non‐self (and thus will not cleave the prokaryotic cell's own DNA).^36^

In 2012, Jennifer Doudna, Emmanuelle Charpentier, and others on their team engineered a synthetic chimera of the tracrRNA and crRNA (now known as single guide RNA, or sgRNA), which was able to direct Cas9 to create a targeted, site‐specific double‐stranded break.^37^ By 2013, investigators had established that the CRISPR‐Cas9 was an effective, facile, and multiplexable method of editing the human genome.^38^, ^39^, ^40^, ^41^

Newer CRISPR‐based editing methods do not reply on unpredictable NHEJ or donor DNA. For example, endonuclease‐deficient Cas proteins can be fused to base‐editing enzymes;^42^ first described in 2016, researchers have recently reported a high‐fidelity base editor with no off‐target mutations (OTMs).^43^ Epigenetic techniques are also being explored using CRISPR‐Cas technology,^44^, ^45^ including linking endonuclease‐deficient Cas proteins to effector molecules. And prime editing addresses genetic disorders caused by multibase variances (such as sickle‐cell and Tay‐Sachs); in this case, the impaired Cas9 is fused to an engineered reverse transcriptase.^46^

ZFNs and TALENs do maintain some advantages: CRISPR requires a PAM sequence, and sgRNA spacer sequences are usually only about 20 base pairs, meaning an inherently reduced targeting capacity (though researchers have recently begun exploring the effects of increased sgRNA length on cleavage efficiency and target specificity^47^). CRISPR vectors are also necessary larger, making delivery more difficult. Overall, however, CRISPR is generally the preferred method of genetic and epigenetic manipulation, especially as improvements are made to the technology. CRISPR’s main advantage over its predecessors lies in the fact that rather than a complex protein as the DNA recognition molecule, the CRISPR system relies on a guide RNA. CRISPR kits are thus significantly cheaper, easier, and more efficiently produced than either ZFNs or TALENs.

### 1.2 Gene selection

Genetic selection happens in nature—natural selection is the mechanism that drives Darwinian evolution. Humans have also been practicing artificial selection for thousands of years, selecting for phenotypic traits when breeding plants and animals. New technologies have been developed over the last 53 years that allow selection of an embryo based on various criteria such as sex, ploidy, and polymorphisms.

#### 1.2.1 Preimplantation genetic testing

Preimplantation genetic testing (PGT) encompasses various techniques used to screen embryos prior to transfer. Originally all referred to as preimplantation genetic diagnosis (PGD), there are actually three types of PGT: aneuploidy detection, now called PGT‐A; monogenic disorder detection, now called PGT‐M; and structural rearrangement detection, now called PGT‐SR.

Preimplantation genetic testing was ideated eleven years before the birth of the first in vitro fertilization (IVF) baby in 1978. Rabbit blastocysts were stained and observed using a fluorescence microscope; screening for sex chromatin allowed for the identification of the female embryos.^48^ Because of the mutagenic potential of the preparation, the embryos were not implanted; a year later, cells from the trophoblasts of rabbit blastocysts were stained and sorted for sex, and the biopsied embryos transferred and allowed to grow to full term (at which point sex was confirmed).^49^

Researchers then began to explore various methods of extracting a single embryonic cell for PGT: a blastomere biopsy (BB) removed during cleavage stage,^50^ trophectoderm biopsy (TB),^51^ and polar body biopsy.^52^ Meanwhile, polymerase chain reaction (PCR) was developed in 1985 and quickly recognized as a potential tool for PGT when it was used to amplify the portion of the β‐globin locus that includes the *Dde*I site (absence of which is diagnostic for sickle‐cell anemia).^53^ The blastomere biopsy technique and PCR were brought together in 1990 when two human pregnancies were established using sex selected embryos to eliminate the risk of inheriting recessive x‐linked conditions.^54^

Fluorescence in situ hybridization (FISH) was the first cytogenetic technique to be used for PGT. Fluorochrome‐labeled site‐specific probes were hybridized to sample DNA, revealing aneuploidy and translocations; in 1993, two laboratories used FISH to identify X‐chromosomes, Y‐chromosomes, and aneuploidy.^55^, ^56^ However, the technique was limited by the number of chromosomes that could be assessed and by its inability to detect monogenic disorders.

Researchers then turned to comparative genomic hybridization (CGH) in 1999.^57^, ^58^ CGH can be thought of as competitive FISH: Sample and reference DNA are each labeled with a different color fluorophore, denatured, and allowed to hybridize to a metaphase spread. The DNA is then microscopically analyzed for differences in fluorescence intensity, indicating copy‐number variation (CNV).

While it was a vast improvement over its predecessor, CGH was time‐consuming (requiring embryos to be freeze‐thawed), labor‐intensive, and limited in its sensitivity. The next generation of CGH technology, array CGH (aCGH), addressed these limitations.^59^ Like traditional CGH, aCGH allows for 24‐chromosome analysis; however, rather than human observation, fluorescence intensity evaluation is performed by a computer, locus by locus, with high specificity and resolution.

A number of other cytogenetic techniques for comprehensive chromosome screening (CCS) have since been developed: digital PCR (or dPCR, wherein a sample‐containing PCR solution is separated into tens of thousands of droplets and the reaction occurs separately in each partition), which can detect CNV, aneuploidy, mutations, and rare sequences; quantitative real‐time PCR (qPCR), in which a preamplification step prior to real‐time PCR allows for rapid detection of aneuploidy in all 24 chromosomes; single nucleotide polymorphism (SNP) array (which involves hybridizing fluorescent nucleotide probes to sample DNA and comparing the resulting fluorescence to a bioinformatic reference), which can detect imbalanced translocation, aneuploidy, and monogenic (and some multifactorial) disease; and next‐generation sequencing (NGS), the high‐throughput, massively parallel DNA sequencing technologies that allow for significantly quicker and cheaper sequencing than the Sanger method and make it possible to screen for everything from SNPs to aneuploidy.

Researchers and IVF laboratories use different combinations of FISH and/or the various CCS techniques.

#### 1.2.2 Other prenatal testing

Often, IVF is not feasible, necessitating postimplantation prenatal testing (when indicated by family history and other risk factors). Amniocentesis, chorionic villus sampling (CVS), and percutaneous umbilical cord sampling (PUBS) were initially paired with karyotyping, which can detect sex, aneuploidy, and some types of structural chromosomal disorders. Karyotyping was superseded by chromosomal microarray techniques (aCGH and SNP array) and, more recently, low‐pass genome sequencing, as these technologies allow detection of CNVs as well as aneuploidy.^60^

Amniocentesis is a procedure in which an ultrasound‐guided needle is inserted transabdominally in order to aspirate amniotic fluid. Applications of amniocentesis extend beyond genetic testing, such as assessment of fetal lung maturity, detection of Rh incompatibility, and decompression of polyhydramnios (accumulation of amniotic fluids).

Prior to 15 weeks’ gestation, the prenatal testing method of choice is CVS, a technique that involves analysis of samples taken from placental tissue. The CVS procedure is ultrasound‐guided and can be performed either transabdominally or transcervically (associated with higher miscarriage rates). CVS carries the risks of miscarriage, amniotic fluid leakage, and limb reduction defects and is limited by the possibility of placental mosaicism and maternal cell contamination.

Percutaneous umbilical cord sampling is a rarely used procedure, performed between 24 and 32 weeks’ gestation, in which fetal blood from the umbilical cord is obtained. Because of the high potential for complications, PUBS is generally reserved for cases in which the pregnancy is deemed high‐risk for genetic disorders and other testing methods (amniocentesis, CVS, and ultrasound) are unable to provide the needed information or have been inconclusive. PUBS is also used to provide more information about fetal health (such as blood gas levels and infection).

In 1997, the presence of cell‐free fetal DNA (cffDNA) in maternal blood was established using PCR amplification with Y‐chromosome probes.^61^ This led to the development of noninvasive prenatal testing (NIPT) of cffDNA. NIPT has been shown to be an accurate and sensitive technique for the detection of some aneuploidies (such as trisomy 21^62^), less so for others.^63^ Because cffDNA comes from the placenta, placental mosaicism can result in inaccurate results. Further, NIPT detects all cell‐free DNA in the mother's blood, including her own; maternal mosaicism or malignancies can also contribute to inaccuracies. As such, NIPT is considered a screening test, rather than a diagnostic test.

## 2 ETHICS OF GENE EDITING

On November 25, 2018, news broke that Jiankui He of Southern University of Science and Technology in Shenzhen, China had registered a clinical trial in which he planned to implant human embryos which had been modified using CRISPR‐Cas9.^64^ Within days, the world learned that not only had edited embryos been implanted, two baby girls, Lulu and Nana, had already been born.^65^

He used CRISPR‐Cas9 to create a nonspecific sequence alteration in the *CCR5* gene. CCR5 is a seven‐transmembrane–spanning G protein–coupled CC chemokine (β chemokine) receptor. When expressed on the surface of a human T cell, CCR5 is the main coreceptor (along with CD4) for the human immunodeficiency virus (HIV). A naturally occurring 32–base pair deletion (with heterozygote allele frequencies of about 10% in people with European origin), known as *CCR5∆32*, has been shown to disable the protein;^66^ heterozygosity of the *CCR5∆32* allele has been shown to slow disease progression, while homozygosity significantly increases disease resistance. He's goal was to knock out *CCR5*, with the desired outcome of creating HIV‐resistant babies (it should be noted that HIV infection in *CCR5∆32^+/+^* individuals has been increasingly reported, associated with X4‐trophic HIV strains—that is, strains that rely exclusively on coreceptor CXCR4 for endocytosis, rather than CCR5^67^).

He presented the details of his investigation^68^ at the Second International Summit on Human Genome Editing, being held “to discuss scientific, medical, ethical, and governance issues associated with recent advances in human gene‐editing research.”^69^ While his manuscript describing the trial was not accepted by any publications, excerpts are available to the public, and various media outlets (and some experts) have been able to view the paper and supplementary data in their entirety. Enough is now known about He's work that it can serve as the basis of a conversation about the ethics surrounding germline gene editing. There are a number of issues—those inherent in the technologies themselves, as well as scientific hurdles that need to be overcome—before initiating clinical trials, to ensure that they are carried out as ethically as possible.

### 2.1 Not all sequence variations are created equally

*CCR5∆32* has been researched extensively, but is one of only a few *CCR5* variants studied. In his abstract, He claims that his team has reproduced this natural variant, but this is not the case: Two embryos were implanted, one of which (Nana) had frameshift mutations on both alleles (a 1–base pair insertion and a 4–base pair deletion, respectively) and the other of which (Lulu) showed a 15–base pair deletion on only one allele. Frameshift mutations have a high probability of disrupting protein structure (and thus function). The 15–base pair deletion, however, will result in five missing amino acids when the protein is translated, and its effect on the protein's function is unknown. He's team could have frozen the embryos, duplicated the sequence alterations in other cell lines, and tested whether or not the genetic changes actually conferred disease resistance, *before* actually implanting the embryos, but it does not appear that they made an effort to fully understand the actual effect of the alterations they had made.^64^ With all of the risks associated with the CRISPR editing process, embryos should not be implanted if the scientists are unsure of the effects.

### 2.2 Mosaicism

A CRISPR‐Cas vector is inserted into a zygote soon after fertilization. If the CRISPR‐induced mutagenesis only occurred during the single‐cell stage, each successive round of cleavage would yield genetically identical cells. However, while the half‐life of the Cas proteins themselves may not be long, the vectors will remain and continue to be transcribed for days. During this time, the embryo will continue to divide, eventually forming a blastocyst of a few hundred cells. Uneven distribution of the plasmid and the RNPs means that there is a significant potential for mosaicism.

In He's laboratory, three to five cells were removed from each blastocyst, and their genomes sequenced. If Lulu's embryo were made up of identical cells (with one wild type allele and one with a 15–base pair deletion) as He had reported, the Sanger chromatogram should have shown two sets of peaks, approximately the same height. However, it appears likely that there were actually three different combinations of alleles: two normal copies, one normal copy and one with a 15–base pair deletion, and one normal copy and an unknown large insertion. Similarly, while Nana's embryo should have shown two alterations, the Sanger chromatogram revealed three.^70^

The suspicion of mosaicism is borne out when sequencing of samples from the cord blood, umbilical cords, and placentas are reviewed. Just as with the embryo sequencing, rampant mosaicism is evident. It is reasonable to assume that the girls’ bodies are mosaic as well, but for an unknown reason, He's team did not test any cells from the girls themselves.^70^ There is therefore the possibility that not all of the cells in Nana's body will have modifications to both *CCR5* alleles, meaning it is possible that Nana is not actually resistant to HIV.

Mosaicism can have myriad effects: Even a few mutated cells in an organ can cause disease, a single cell can develop into a tumor, and any allelic variation in germ cells will be inherited by the following generation. There is no way to sequence a cell's genome without destroying the cell itself; as such, it is currently impossible to rule out mosaicism in a blastocyst.

### 2.3 Off‐target effects

As efficient as CRISPR is, there is a high probability of OTMs. He's team reported that in addition to the *CCR5* gene edits, there was only one OTM, a 1–base pair insertion in a noncoding region of Chromosome 1 in Lulu's genome. This was based on their relatively limited sequencing, however; as noted above, mosaicism cannot be ruled out. (It should also be noted that there were flaws in the sequencing itself, so there may be other alterations that were missed in the screening, on top of the mosaicism.^70^)

### 2.4 Other consequences of target gene modification

When undertaking to knock out a gene in an embryo, it is vital to understand all of the functions of that gene.

CCR5 is a chemokine receptor that mediates leukocyte chemotaxis, and thus helps mount immune response. It is therefore unsurprising that homozygosity for the *CCR5∆32* variant has been shown to be significantly correlated with more symptomatic infection and higher mortality rates in patients with West Nile virus,^71^ influenza A,^71^, ^72^ and tick‐borne encephalitis.^73^ It has also been shown to be associated with upregulation of certain CC chemokine ligands, and in turn associated with progressive reduction in survival time for patients with multiple sclerosis (MS).^74^ Is it ethical to create a sequence variation that confers resistance to one illness, while increasing the likelihood of succumbing to another?

Public health conversations will need to change as well. It is possible, for example, that some with a *CCR5* edit will engage in riskier sexual practices, or that some with a *PCSK9* edit (which is associated with decreased levels of low‐density lipoprotein cholesterol, or LDL, in the blood^75^, ^76^) will be less likely to make behavioral changes such as increased exercise and diet modification.

### 2.5 Which genes/diseases to target?

Many who have viewed He's work have questioned why he chose to focus on *CCR5* and HIV resistance. HIV prevalence in China is relatively low,^77^ and current treatments can keep viral loads at almost undetectable levels. He stated that his research could help tamp down the HIV/AIDS epidemic; the most hard‐hit areas (such as Africa), however, would likely not gain much benefit from gene‐editing technologies.

Per a December 2018 poll,^78^ Americans draw the line at so‐called enhancement, but favor the use of genetic engineering to address disease and disability. Which diseases and disabilities to target, however, is still an open discussion. Some questions that may help inform that decision: Should there be a focus on infectious disease resistance? Only fatal conditions? Will we decide that there is a need to quantify the degree of suffering? If an effective treatment already exists, should we still seek prevention through genetic modification? Is childhood versus adulthood onset of illness an important factor? Not all sequence variants are guaranteed to cause disease (eg, *BRCA* genes); should they be considered? What about orphan diseases? And should certain types of disabilities be prioritized over others?

### 2.6 OTHER ISSUES

#### 2.6.1 Clinical research ethics

The history of research using human subjects has been blemished by unethical treatment of the subjects themselves. From Imperial Japan to the Tuskegee Institute, examples of atrocities committed in the name of medical science can be found across the world. As a result, a number of guidelines have been developed to facilitate ethical research going forward.^79^ Nazi Germany engaged in abominable human experimentation during World War II; in 1947, the Nuremberg Military Tribunal (during which Nazi physicians and administrators were tried for war crimes and crimes against humanity) resulted in the Nuremberg Code, a statement aimed at preventing such abuses in the future. Stemming from a reaction to the same offenses, the World Medical Association produced *Ethical Principles for Medical Research Involving Human Subjects*—known as the Declaration of Helsinki—in 1964 (it has been modified a few times since), which in 1982 was adapted by the Council for International Organizations of Medical Sciences into a manual, *Proposed International Ethical Guidelines for Biomedical Research Involving Human Subjects*, as a guideline for World Health Organization (WHO) member countries. (Individual countries have created their own guidelines, as well. In the United States, for example, the National Research Act of 1974 established the National Commission for the Protection of Human Subjects of Biomedical and Behavioral Research—often referred to as the Belmont commission—which issued the 1979 *Ethical Principles and Guidelines for the Protection of Human Subjects of Research*, now known as the Belmont Report. The National Research Act of 1974 also established a set of regulations regarding human subject research; by 1991, after various updates and additions, the government decided that the regulations should become a “Common Rule” covering all federally connected research, codified in Title 45, Part 46 of the Code of Federal Regulations.)

This article will not delve into all of He's ethical missteps as regards his research (such as the fact that he registered the trial with the Chinese Clinical Trial Registry in November 2018, after the twins had already been born). However, there are a few key issues, directly addressed by at least one of these major reports, which can be considered in terms of guiding future ethics discussions regarding gene‐editing clinical trials.

First and foremost, clinical trial participants should be informed of all of the associated risks and benefits. There is significant ambiguity as to the informed consent process in He's study. First, the experiment was misleadingly couched as an HIV vaccine trial.^65^, ^80^ It is also unclear how much the parents in He's study understood about risks such as mosaicism or increased susceptibility to other infections. The investigator is responsible for keeping participants informed throughout the study; it does not appear that in this case they were informed of the mosaicism present in both embryos (in his presentation at the human genome editing summit, He said only that the parents had been informed about one OTM^68^ and does not even mention the mosaicism in his manuscript^70^). It is likely that were the parents fully informed, they would have decided not to implant one or both embryos. This raises another question: Should parents be the ones to make such a decision, or is that the responsibility of the researchers and doctors?

The selection of the study participants is an issue, as well: The first inclusion criterion is that the participants must be a married couple wherein the husband is HIV‐positive and the wife is HIV‐negative.^81^ Father‐to‐child transmission of HIV is rare (especially when the father is on antiretroviral therapy and the mother is on preexposure prophylaxis), but it is possible; for this reason, many couples opt for sperm washing (to separate the sperm from the virus), followed by either in vitro fertilization (IVF) or intrauterine implantation (IUI). While there is no explicit law against HIV‐positive parents accessing these procedures, it is unlikely that it would be approved by hospitals’ ethics committees.^82^ Chinese couples often travel to other countries (such as Thailand) for the procedure, but it can cost hundreds of thousands of yuan.^83^ The couples selected for He's study may have seen participation as their only chance to have children—indeed, He described the father as having “lost hope for life.”^68^ This makes them particularly vulnerable to exploitation. (This may also have informed their decision to implant both embryos rather than just the one that had alterations to both alleles.)

Study participants should be able to voluntarily withdraw from the research at any point; He's informed consent form stipulates that were the couple to withdraw from the study (at any point between the implantation of the embryo in the first IVF cycle and 28 days postbirth), they would be responsible for reimbursing the laboratory for all project costs (and that if reimbursement was not received within 10 calendar days of withdrawal, a substantial fine—more than the average annual income of a Chinese citizen—would be imposed).^80^ This is sufficiently cost prohibitive as to prevent a subject from withdrawing from the study.

It is unclear whether He's research actually underwent an ethics review process: In his manuscript, He claims that the Medical Ethics Committee of the Shenzhen Harmonicare Women's and Children's Hospital approved the study in March 2017, but only elaborates by stating that his team was “… told that the committee held a comprehensive discussion of risks and benefits… During the study, the director of the ethics committee was constantly updated about the state of the clinical trial.”^84^ The hospital has since denied that the study was reviewed at all, and claims that the signatures on the approval were forged.^85^ What is clear is that a number of regulations were violated or circumvented, including the guidelines for embryo research which allow an edited embryo to be cultured for no more than 14 days and prohibit its implantation,^86^ as well as the aforementioned limitations on assisted reproductive services for HIV‐positive parents.^82^ It is likely that He switched blood samples and kept many of the IVF technicians and obstetricians in the dark as to the nature of the study to get around these issues.^87^

Finally, it is reasonable to consider whether He was qualified to be the investigator on such a trial: He had published one paper about CRISPR (in 2010, before human gene editing was an application of the technology), his background was in physics (he crossed over into biophysics), and he had no medical training. This is especially concerning as biohackers have made available both the equipment and the basic blueprints for home CRISPR editing (see the Other Perspectives section)—including advice on how to obtain human embryos and eventually implant them.^88^

#### 2.6.2 Socioeconomic disparities

Multiple polls have shown that the majority of people around the world are opposed to the use of genetic engineering of embryos for enhancement, such as athletic ability and intelligence, or for altering physical characteristics, such as eye color and height.^89^ It is easy to conceive of the risk of a new age of eugenics.

But even the application of genetic modification to address medical needs holds the potential for establishing inequality. The technology will remain incredibly expensive for some time, prohibitively so for most people. *CCR5* edits lie in an ill‐defined area between medical need and enhancement; an unfair health advantage will be established if such modifications are only accessible to the wealthy. Other kinds of edits may mean the difference between life and death; should potentially life‐saving therapies only be available to those with financial means? Put another way, should those individuals on one side of the growing socioeconomic gap be the only ones protected from the suffering that comes with illnesses such as Alzheimer's disease, Huntington disease, or cystic fibrosis?

#### 2.6.3 Possible stigma

Especially while the concept is still novel, it is difficult to predict how society will feel about gene‐edited babies. Will Nana and Lulu face any sort of backlash? Conversely, if and when gene editing becomes commonplace, will there be a stigma associated with *not* having been edited in some way, such as still being susceptible to various infectious diseases? Might children like Lulu be less accepted for not carrying a desired modification? He wanted to spare HIV‐infected individuals’ children the stigma and discrimination their parents endured;^90^ it is possible that having edited genes has replaced one potential stigma with another.

#### 2.6.4 Insurance

Because gene editing will be a tool to cure and prevent illness, insurance coverage will be an important part of the conversation. First, will insurance cover the editing itself? If so, will germline versus somatic cell editing be an important distinction? Will coverage be based on the targeted illness or disability (and expected associated costs)? And who will decide which edits are considered medically necessary and which are considered elective?

Once babies born from edited embryos are born, more questions arise. Will those whose genes have not been edited to prevent certain illnesses be considered to have preexisting conditions? Will they be expected to pay more for coverage? On the other side of the coin, will those who have had their genes edited (especially when the technology is first rolled out) pay more because of possible off‐target risks or potential negative consequences of editing (eg, the increased susceptibility to influenza associated with *CCR5* editing)?

#### 2.6.5 Other perspectives

A full discussion of ethics requires a balanced presentation of various points of view.

There are those who object to continued research into gene editing, especially in zygotes, for myriad reasons. For example, some feel that gene editing is “playing God” and that it is not man's role to make changes to the basic building blocks of humanity; others are concerned about the potential that the technology, once perfected, could be co‐opted to produce designer babies; there is the consideration that opening a market for human eggs for research could lead to exploitation of disadvantaged women; and still others have concerns similar to those who are opposed to embryonic stem cell research—such as the conviction that embryos should not be created for the purpose of research, or that un‐implanted embryos (which they consider potential life) should not be destroyed.

There are also those who believe that not only should research continue, but that even nascent technology such as gene editing should be accessible to the public.^91^ Known as biohackers, these scientists and activists laud the efforts like Jiankui He's.^88^ Educational and laboratory materials are currently available to essentially anyone. It is even possible to purchase CRISPR kits,^92^ and while a new California law requires that such kits are labeled “not for self‐administration”^93^ there are currently no laws prohibiting people from doing just that—in fact, the owner of one company was investigated by the California Medical Board for unlicensed practice of medicine after injecting himself with CRISPR, but the investigation was dropped after four months with “no further action… anticipated.”^94^

## 3 GLOBAL DISCUSSIONS ON GERMLINE EDITING

While it is impossible to mandate that all countries follow the same set of guidelines, it is possible to establish guiding principles for the risk‐benefit analyses and ethical discussions each country will undertake in developing their own regulatory framework. Because science moves faster than regulation, the scientific community as a whole can also use these principles to help guide ethically charged research decisions where no regulations yet exist. To that end, various groups have been meeting all over the world to try to come to a consensus on how to proceed with germline editing research and the potential clinical applications thereof.

### 3.1 Before He’s announcement

In 2015, the International Bioethics Committee (IBC), part of the United Nations Educational, Scientific, and Cultural Organization (UNESCO), released the Report of the IBC on Updating its Reflection on the Human Genome and Human Rights.^95^ The report considers other technologies as well, but “recommends a moratorium on genome editing of the human germline.”

Also in 2015, investigators in China announced that they had successfully used CRISPR to edit a nonviable human embryo.^96^ This inspired the first International Summit on Human Gene Editing, held December 2015 in Washington, D.C. Hosted by the US National Academy of Sciences, US National Academy of Medicine, the Royal Society of the UK, and the Chinese Academy of Sciences, the summit brought together more than 3500 stakeholders (500 in person and 3000 online) from around the world to discuss human gene editing. At the end of the summit, the organizing committee released a statement advising ongoing global engagement and discussion, and outlined their conclusions regarding gene editing:^97^ “(i)ntensive basic and preclinical research is clearly needed and should proceed, subject to appropriate legal and ethical rules and oversight…”; “(m)any promising and valuable clinical applications of gene editing are directed at altering genetic sequences only in somatic cells… [and] they can be… evaluated within existing and evolving regulatory frameworks for gene therapy…”; and “(g)ene editing might also be used, in principle, to make genetic alterations in gametes or embryos…” The statement goes on to address the ethical, legal, and scientific questions surrounding germline editing that have yet to be answered, and warns:

It would be irresponsible to proceed with any clinical use of germline editing unless and until (a) the relevant safety and efficacy issues have been resolved, based on appropriate understanding and balancing of risks, potential benefits, and alternatives, and (b) there is broad societal consensus about the appropriateness of the proposed application. Moreover, any clinical use should proceed only under appropriate regulatory oversight. At present, these criteria have not been met for any proposed clinical use: the safety issues have not yet been adequately explored; the cases of most compelling benefit are limited; and many nations have legislative or regulatory bans on germline modification. However, as scientific knowledge advances and societal views evolve, the clinical use of germline editing should be revisited on a regular basis.

While this statement in no way gives a green light for trials such as He's, it also does not call for an outright moratorium. In March 2017, another Chinese team published the results of the first use of CRISPR in viable human embryos.^98^ Less than two years later, He's work was revealed to the world.

### 3.2 After He’s announcement

The article about He's trial was published the day before the second International Summit on Human Gene Editing. As they had at the first summit, organizers released a concluding statement on the last day. Surprisingly, not only does the statement again fall short of calling for a moratorium on clinical use of gene editing, the language is even softer than that of the first summit statement:

The variability of effects produced by genetic changes makes it difficult to conduct a thorough evaluation of benefits and risks. Nevertheless, germline genome editing could become acceptable in the future if these risks are addressed and if a number of additional criteria are met. These criteria include strict independent oversight, a compelling medical need, an absence of reasonable alternatives, a plan for long‐term follow‐up, and attention to societal effects. Even so, public acceptability will likely vary among jurisdictions, leading to differing policy responses.

The organizing committee concludes that the scientific understanding and technical requirements for clinical practice remain too uncertain and the risks too great to permit clinical trials of germline editing at this time. Progress over the last three years and the discussions at the current summit, however, suggest that it is time to define a rigorous, responsible translational pathway toward such trials.

In December 2018, seeing the need for a more substantial framework of regulatory guidance, the WHO established the Advisory Committee on Developing Global Standards for Governance and Oversight of Human Genome Editing, “a global, multi‐disciplinary expert panel to examine the scientific, ethical, social and legal challenges associated with human genome editing… tasked to advise and make recommendations on appropriate institutional, national, regional and global governance mechanisms for human genome editing.”^99^ They have established the Human Genome Editing Registry to collect information on human clinical trials involving genome editing, and the WHO has supported the advisory committee's interim recommendation that “it would be irresponsible at this time for anyone to proceed with clinical applications of human germline genome editing.”^100^

In 2019, the US National Academies of Medicine and Science, together with the Royal Society, convened the International Commission on the Clinical Use of Human Germline Genome Editing. The goal of commission is:^101^

… with the participation of science and medical academies around the world, to develop a framework for scientists, clinicians, and regulatory authorities to consider when assessing potential clinical applications of human germline genome editing. The framework will identify a number of scientific, medical, and ethical requirements that should be considered, and could inform the development of a potential pathway from research to clinical use—if society concludes that heritable human genome editing applications are acceptable.

The commission's final report is scheduled to be released in the spring of 2020.

As the science progresses, there are clearly significant conversations yet to be had.

## CONFLICT OF INTEREST

The authors have stated explicitly that there are no conflicts of interest in connection with this article.
